# Musculoskeletal adverse events reported post-hepatitis B vaccination in the vaccine adverse event reporting system

**DOI:** 10.3389/fpubh.2025.1560973

**Published:** 2025-05-02

**Authors:** Yiqing Sun, Bukun Zhu, Xiang Li, Zhanyang Luo, Weiguo Bian

**Affiliations:** ^1^Department of Orthopedics, The First Affiliated Hospital of Xi’an Jiaotong University, Shaanxi, China; ^2^Department of Infection, Longhua Hospital, Shanghai University of Traditional Chinese Medicine, Shanghai, China; ^3^School of Medicine, Xiamen University, Fujian, China; ^4^Department of Pharmacy, Shanghai Pudong Hospital, Fudan University Pudong Medical Center, Shanghai, China

**Keywords:** HBV, hepatitis B, vaccine, VAERS, musculoskeletal

## Abstract

**Introduction:**

Hepatitis B virus (HBV) is a major cause of chronic liver disease. While the hepatitis B vaccine has been proven effective in preventing HBV infection, concerns regarding Events Supposedly Attributable to Vaccination or Immunization (ESAVI) persist. This study aims to utilize the Vaccine Adverse Event Reporting System (VAERS) database to explore potential associations between the hepatitis B vaccine and musculoskeletal system AEs, providing a scientific basis for vaccine safety evaluations.

**Methods:**

This study analyzed VAERS data from 1990 to 2024, focusing on 76,887 reports associated with hepatitis B vaccines. Disproportionality analysis methods, such as the Proportional Reporting Ratio (PRR) and Reporting Odds Ratio (ROR), were applied to identify the distribution and signal strength of musculoskeletal-related AEs. Furthermore, multivariable logistic regression analysis was conducted to explore association between patients with HBV vaccine and death.

**Results:**

Musculoskeletal system ESAVIs constituted a significant portion of all reports, including tendon fibrosis (ROR = 251.82), myofascitis (ROR = 107.51), fasciitis (ROR = 71.52), and osteoarthritis (ROR = 7.56). Tendon fibrosis demonstrated the strongest association, potentially linked to chronic inflammatory responses and abnormal tissue repair induced by aluminum adjuvants. Most AEs occurred within 30 days post-vaccination, though some, such as myofascitis, had a longer mean onset time (1,671 days), reflecting the slow-release properties of aluminum adjuvants. In multivariable logistic regression analysis, we concluded that male and combination vaccine treatment were risk factors while age from18-64 years was a protective factors of death.

**Conclusion:**

This study identifies potential associations between hepatitis B vaccination and musculoskeletal system AEs, emphasizing the need for thorough pre-vaccination assessments and post-vaccination monitoring for high-risk individuals.

## 1 Introduction

Hepatitis B virus (HBV) is a major cause of chronic liver disease worldwide, affecting approximately 296 million people globally (1). As an effective preventive measure, the hepatitis B vaccine has been widely used to combat HBV infection and is considered a priority vaccine under the World Health Organization’s (WHO) Global Immunization Program. However, with the increasing coverage of vaccination, concerns about vaccine-related safety have also risen. Although the hepatitis B vaccine is widely regarded as safe, with most adverse events being benign, mild, and transient, increasing public concerns over rare and severe adverse events warrant comprehensive safety evaluations.

While pre-licensure clinical trials provide initial evidence of vaccine safety, the limited scale and selective populations of these trials may fail to identify rare Events Supposedly Attributable to Vaccination or Immunization (ESAVI) or those more common in specific subpopulations (2). To address this limitation, the Vaccine Adverse Event Reporting System (VAERS) in the United States continuously collects and analyzes post-vaccination AE data through a spontaneous reporting mechanism, offering crucial support for post-marketing safety surveillance.

Aluminum-based adjuvants, which are the most widely used adjuvants in vaccines, are present in approximately one-third of currently licensed vaccines (3). Once absorbed into the body, aluminum disperses into various tissues, with the majority accumulating in bones, the liver, lungs, and the nervous system. This issue is particularly critical for individuals with chronic kidney disease, where aluminum cannot be effectively cleared from the body, leading to cumulative effects over time, especially in the skeletal and nervous systems (4). Thus, vaccine-related musculoskeletal system reactions warrant attention.

Although the hepatitis B vaccine has demonstrated favorable safety profiles in most populations, research focusing on musculoskeletal system ESAVIs remains scarce. Existing literature primarily consists of case reports or small-scale analyses, suggesting that vaccination might trigger immune-mediated inflammatory responses or other mechanisms, leading to muscle pain, fasciitis, and other musculoskeletal abnormalities (5–7). However, these conclusions are often based on limited data and lack systematic understanding, with the underlying mechanisms yet to be elucidated. Given the limited research on musculoskeletal system ESAVIs following hepatitis B vaccination, this study aims to utilize the VAERS database to explore the association between the hepatitis B vaccine and musculoskeletal system ESAVIs and investigate potential mechanisms. This will provide a scientific basis for vaccine safety evaluations and reinforce public confidence in vaccination programs.

## 2 Methods

### 2.1 Data source

This study utilized the publicly available VAERS database, a platform developed and operated by the U.S. Centers for Disease Control and Prevention (CDC). VAERS is designed to monitor vaccine safety, detect atypical and rare vaccine-related adverse events, and identify risk factors contributing to these events. The database comprises reports from patients, parents (on behalf of minor patients), healthcare providers, vaccine manufacturers, and regulatory agencies worldwide. To ensure data confidentiality, patient information in the reports is de-identified and anonymized before inclusion in the database. Consequently, the study was exempted from review by the Human Research Ethics Committee at the University of Adelaide.

ESAVI reports in VAERS include patient demographic information, dates of vaccination and ESAVI onset, detailed ESAVI history, clinical history, current medications, comorbidities, and unstructured narratives describing post-vaccination clinical manifestations and ESAVI diagnoses. The ESAVI symptom names cited in this study are encoded using the preferred terms (PTs) from the Medical Dictionary for Regulatory Activities (MedDRA) to standardize the terminology for ESAVIs internationally.

### 2.2 Data screening

The study utilized MedDRA to filter cases where the product name was identified as the hepatitis B vaccine and categorized as the primary suspected drug. Patient information, drug details, and ESAVI data were extracted. Further screening criteria included the exclusion of duplicate and incomplete reports to form the raw dataset for ESAVI analysis. Between January 1990 and October 2024, a total of 76,887 original entries related to the hepatitis B vaccine were recorded in the VAERS database.

### 2.3 Statistical analysis

To identify potential ESAVI signals, four disproportionality analysis methods were applied: the Reporting Odds Ratio (ROR) (8), Proportional Reporting Ratio (PRR) (9), Bayesian Confidence Propagation Neural Network (BCPNN) (10), and Multi-item Gamma Poisson Shrinker (MGPS) (11). ROR is the frequentist method comparing the odds of a specific ESAVI with a given vaccine versus all other vaccines; PRR calculates the proportion of a specific ESAVI among reports for a given vaccine relative to other vaccines; BCPNN is a Bayesian approach used by WHO-UMC to estimate the strength of association based on information components; MGPS is a Bayesian data mining method used by the FDA that adjusts for data sparsity by shrinking observed-to-expected ratios. A signal was considered present if the number of observed cases (a) was ≥3 and the lower bound of the 95% confidence interval (CI) for ROR exceeded 1.

ESAVI signals identified under the condition of a ≥ 3 were coded and categorized using MedDRA Version 23.0 (updated in March 2020) based on system organ class (SOC). The SOC classification groups terms by etiology, site of manifestation, or purpose. Due to the multi-axial structure of MedDRA, a single PT can belong to multiple SOCs; however, each PT has only one primary SOC for classification purposes (12). Using SOC, further analyses were conducted to characterize the distribution of ESAVIs in the population vaccinated with the hepatitis B vaccine.

To investigate factors associated with fatal outcomes (Death), we performed multivariable logistic regression analysis. The independent variables included age, gender and treatment group (HBV vaccine alone and combination treatment). Age was categorized into three groups: <18 years, 18–64 years and ≥65 years. *p*-values less than 0.05 were considered statistically significant.

## 3 Results

### 3.1 Baseline characteristics of adverse event reports among hepatitis B vaccine recipients

**Figure 1:**
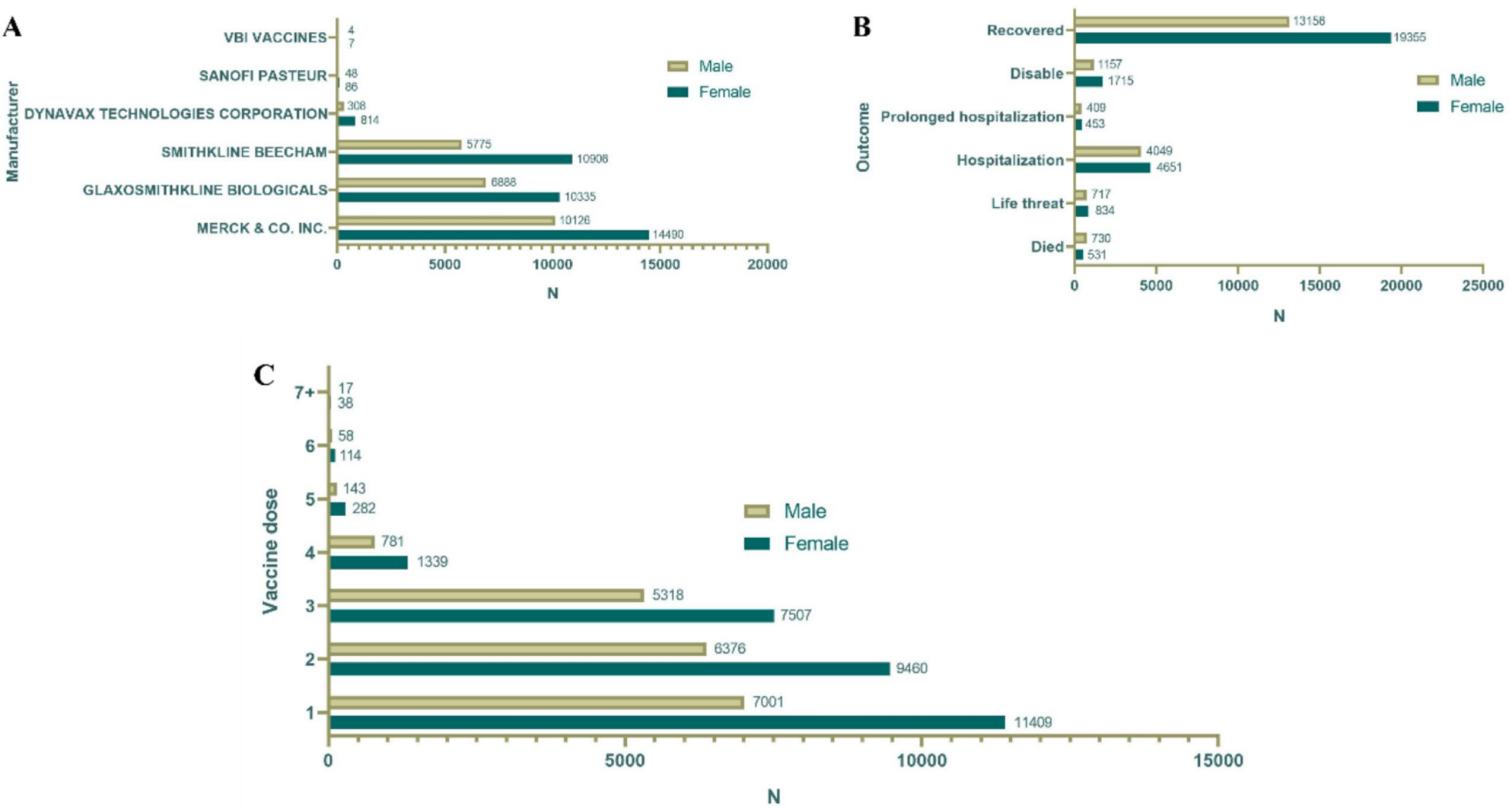
Baseline data of hepatitis B vaccine stratified by sex. **(A)** Distribution of vaccine manufacturers. **(B)** Outcome distribution of vaccine; distribution of vaccine doses. **(C)** Gender distribution of different vaccine dose.

**Figure 2:**
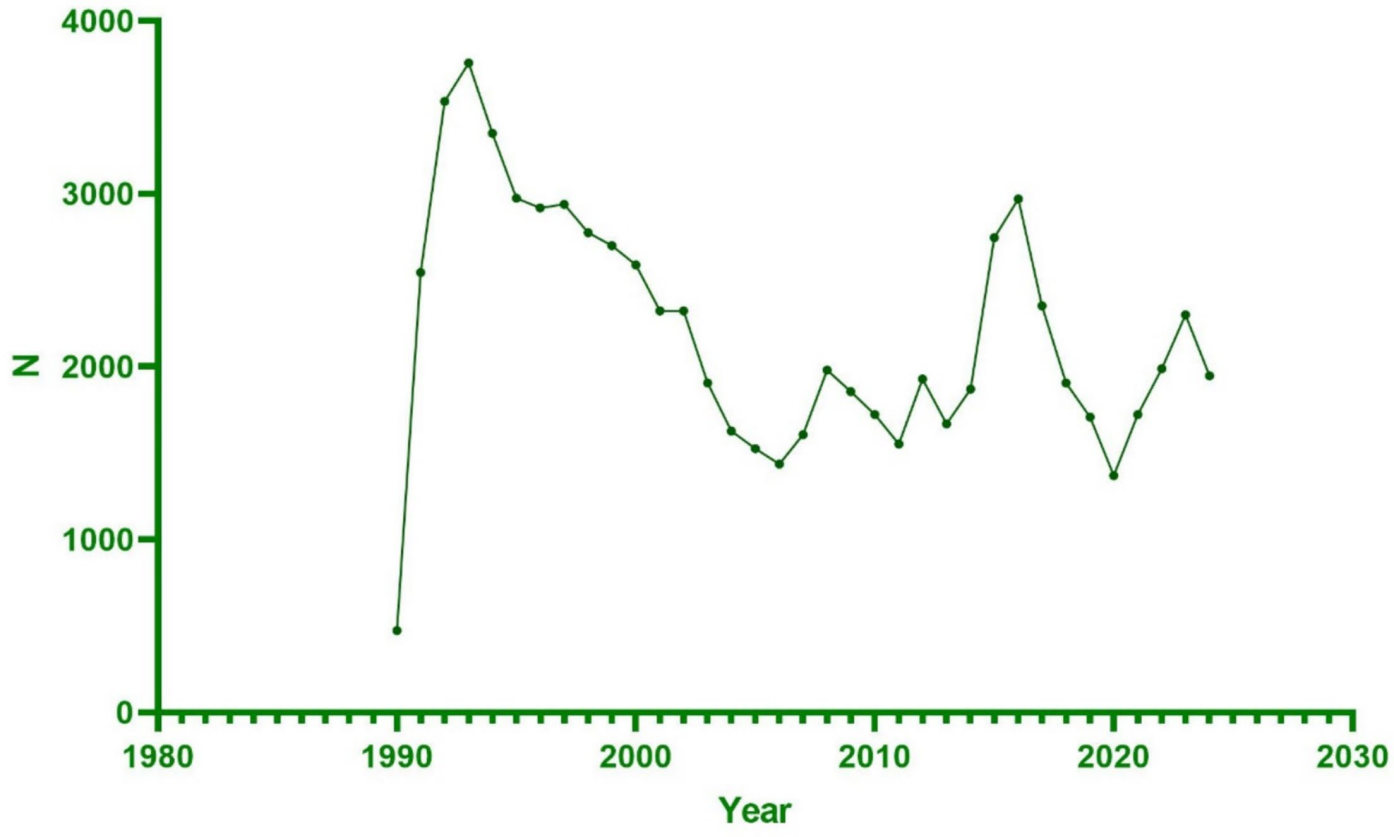
Distribution of hepatitis B vaccine annual reporting frequency in VAERS database.

A total of 76,887 reports were received from hepatitis B vaccine recipients. The vaccine manufacturers involved included MERCK & CO, GlaxoSmithKline Biologicals, SmithKline Beecham, Dynavax Technologies Corporation, Sanofi Pasteur, and VBI Vaccines. Among the states reporting ESAVIs, the top five were foreign state, California, Texas, New York, and Michigan. In terms of gender distribution, females (52.1%) significantly outnumbered males (34.1%). Regarding age, a substantial proportion of recipients were aged <18 years (40.0%) and 18–64 years (36.8%). Between January 1990 and October 2024, the highest number of ESAVI reports was recorded in 1993. Most reports involved recipients receiving one dose (25.3%) or two doses (21.8%) of the vaccine. In terms of severity, 13,354 cases (17.4%) exhibited serious clinical symptoms, including 1,411 deaths, 1,585 life-threatening events, and 2,939 disabilities (Table 1; Figures 1, 2).

**Table 1:** Baseline data of hepatitis B vaccine reported in the VAERS database.

Characteristic	N	Percentage
State		
Foreign state	12,586	16.4%
California	5,269	6.9%
Texas	3,903	5.1%
New York	3,030	3.9%
Michigan	2,878	3.7%
Missing	6,284	8.2%
Age		
<18	30,759	40.0%
18–64	28,297	36.8%
65–84	846	1.1%
≥85	19	0.0%
Unknown	16,966	22.1%
Sex		
Female	40,078	52.1%
Male	26,213	34.1%
Unknown	10,596	13.8%
Serious symptom		
No	63,533	82.6%
Yes	13,354	17.4%
Alone medication		
No	31,078	40.4%
Yes	45,809	59.6%
Outcome		
Recovered	33,810	44.0%
Hospitalization	9,421	12.3%
Disable	2,939	3.8%
Life threat	1,585	2.1%
Died	1,411	1.8%
Prolonged hospitalization	907	1.2%

### 3.2 Analysis of musculoskeletal adverse events among hepatitis B vaccine recipients

Based on the system organ class (SOC) classification, musculoskeletal system ESAVIs ranked sixth among all SOCs [ROR (95% CI): 0.89 (0.87–0.90)] and therefore warrant attention (Supplementary Table S1). A total of 16,921 ESAVIs related to the musculoskeletal system were identified. Disproportionality analysis revealed 32 preferred terms (PTs) associated with hepatitis B vaccination. Among these, the top 20 PTs were categorized as follows:

Bone-related PTs (7 types): The top five included arthritis [ROR (95% CI): 3.2 (2.93–3.49)], osteoarthritis [ROR (95% CI): 7.56 (6.76–8.45)], rheumatoid arthritis [ROR (95% CI): 2.45 (2.18–2.76)], arthropathy [ROR (95% CI): 5.18 (4.5–5.97)], and bone disorder [ROR (95% CI): 8.69 (6.73–11.23)]; Muscle-related PTs (11 types): The top five included muscle twitching [ROR (95% CI): 2.34 (2.13–2.56)], myofascitis [ROR (95% CI): 107.51 (86.82–133.12)], fasciitis [ROR (95% CI): 71.52 (57.25–89.35)], fibromyalgia [ROR (95% CI): 3.06 (2.6–3.59)], and muscle atrophy [ROR (95% CI): 4.73 (3.95–5.65)]; PTs involving both bone and muscle: Nuchal rigidity [ROR (95% CI): 16.19 (14.17–18.49)] and systemic lupus erythematosus [ROR (95% CI): 5.82 (5.04–6.73)].

**Figure 3:**
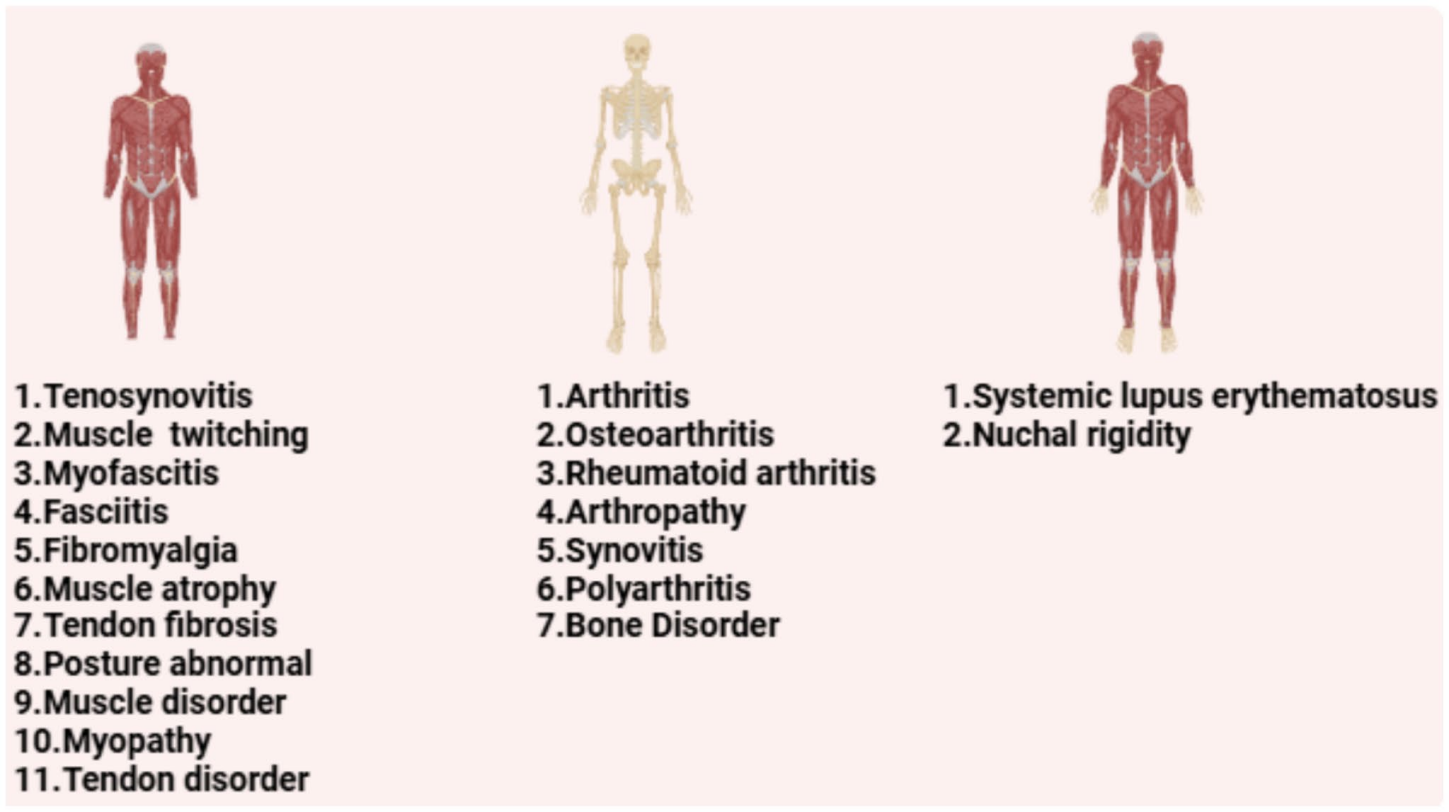
The PT in the musculoskeletal system of hepatitis B vaccine is classified according to muscle, bone and skeletal-muscle.

Based on ROR values, the top five PTs were Fibrosis tendinous [ROR (95% CI): 251.82 (157.34–403.04)], myofascitis [ROR (95% CI): 107.51 (86.82–133.12)], fasciitis [ROR (95% CI): 71.52 (57.25–89.35)], nuchal rigidity [ROR (95% CI): 16.19 (14.17–18.49)], and osteoarthritis [ROR (95% CI): 7.56 (6.76–8.45)]. These findings suggest a higher correlation between muscle-related PTs and ESAVIs in hepatitis B vaccine recipients (Table 2; Figure 3).

**Table 2:** Top 20 signal strength of ESAVI reports for hepatitis B vaccine at the preferred term (PT) level in the VAERS database.

PT	Number	ROR (95%CI)	PRR (χ^2^)	EBGM05	IC025
Arthritis	550	3.2 (2.93–3.49)	3.19 (765.37)	3.02 (2.81)	1.6 (1.47)
Muscle twitching	503	2.34 (2.13–2.56)	2.33 (361.25)	2.26 (2.09)	1.17 (1.04)
Osteoarthritis	367	7.56 (6.76–8.45)	7.55 (1741)	6.47 (5.89)	2.69 (2.53)
Myofascitis	321	107.51 (86.82–133.12)	107.39 (8866.72)	28.88 (24.15)	4.85 (4.64)
Nuchal rigidity	310	16.19 (14.17–18.49)	16.17 (3099.04)	11.65 (10.43)	3.54 (3.36)
Rheumatoid arthritis	288	2.45 (2.18–2.76)	2.45 (232.46)	2.36 (2.14)	1.24 (1.07)
Fasciitis	223	71.52 (57.25–89.35)	71.47 (5391.49)	25.52 (21.18)	4.67 (4.43)
Arthropathy	218	5.18 (4.5–5.97)	5.18 (646.96)	4.68 (4.16)	2.23 (2.02)
Systemic lupus erythematosus	211	5.82 (5.04–6.73)	5.82 (730.62)	5.18 (4.59)	2.37 (2.16)
Fibromyalgia	158	3.06 (2.6–3.59)	3.06 (202.33)	2.9 (2.53)	1.54 (1.3)
Muscle atrophy	136	4.73 (3.95–5.65)	4.72 (355.26)	4.31 (3.72)	2.11 (1.85)
Fibrosis tendinous	132	251.82 (157.34–403.04)	251.71 (4337.14)	33.99 (22.93)	5.09 (4.74)
Posture abnormal	116	2.55 (2.11–3.07)	2.55 (102.18)	2.45 (2.09)	1.29 (1.02)
Muscle disorder	106	4.8 (3.92–5.87)	4.8 (282.88)	4.37 (3.69)	2.13 (1.83)
Myopathy	82	9.15 (7.19–11.64)	9.14 (479.77)	7.57 (6.19)	2.92 (2.57)
Bone disorder	72	8.69 (6.73–11.23)	8.69 (399.03)	7.26 (5.86)	2.86 (2.49)
Polyarthritis	68	2.7 (2.11–3.45)	2.7 (67.9)	2.59 (2.11)	1.37 (1.01)
Tendon disorder	47	3.15 (2.34–4.24)	3.15 (63.72)	2.99 (2.33)	1.58 (1.15)
Synovitis	45	3.83 (2.82–5.21)	3.83 (85.54)	3.57 (2.76)	1.84 (1.39)
Tenosynovitis	36	5.32 (3.76–7.54)	5.32 (110.87)	4.79 (3.58)	2.26 (1.76)

### 3.3 Onset time of adverse events among hepatitis B vaccine recipients

**Figure 4:**
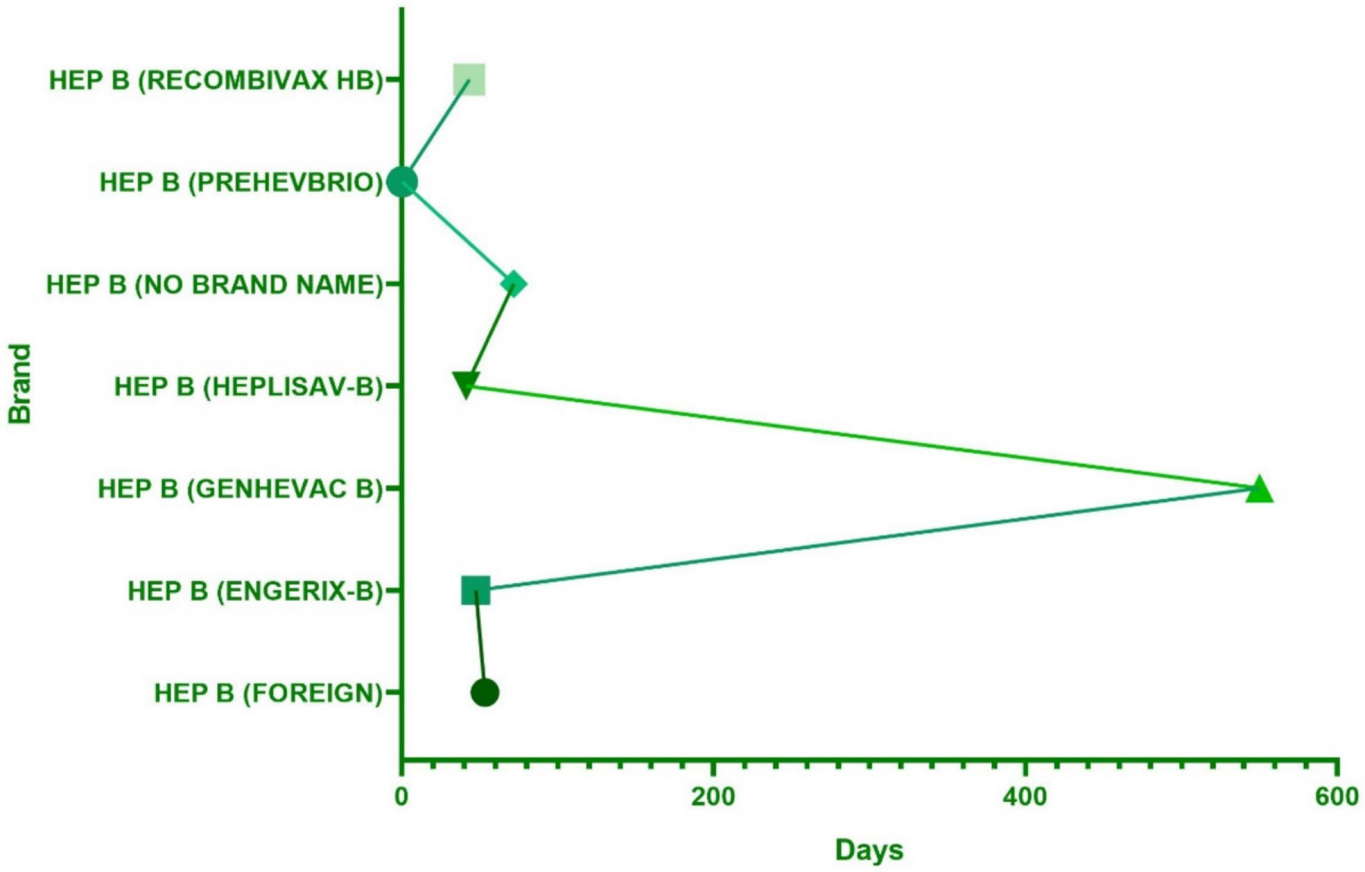
The distribution of hepatitis B vaccine brands in the occurrence time of adverse reactions.

**Figure 5:**
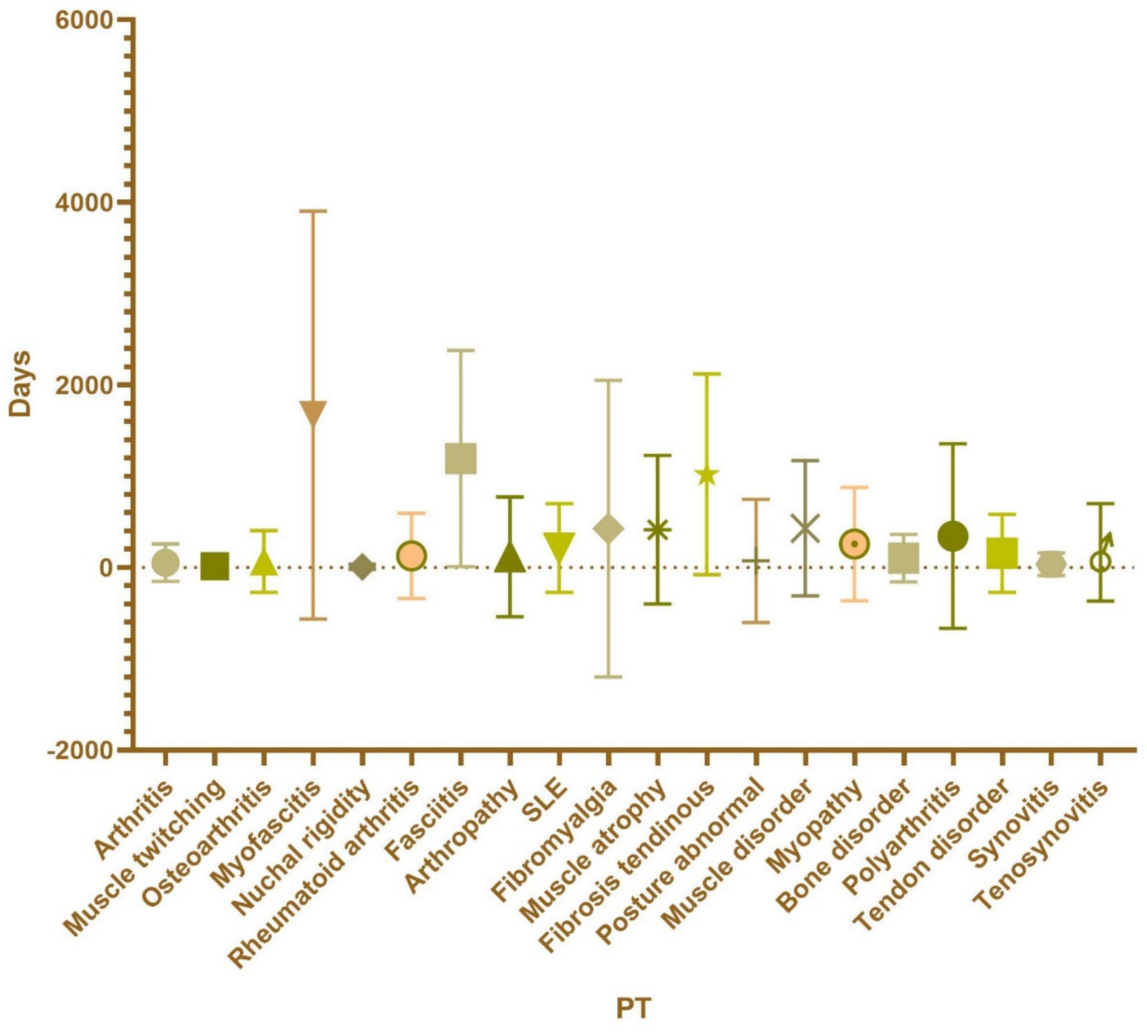
Onset time of ESAVIs of the top 20 PT in musculoskeletal system.

Regarding the onset time of ESAVIs among hepatitis B vaccine recipients, the majority of ESAVIs were acute, occurring within 0–30 days post-vaccination, with 53,466 cases reported. An analysis of whether different vaccine brands influenced ESAVI onset time revealed that HEP B (GENHEVAC B) had the longest mean ESAVI onset time, while HEP B (RECOMBIVAX HB) had the shortest, with most events occurring on day 0. These finding merits further investigation. Focusing on musculoskeletal-related ESAVIs, myofascitis had the longest mean ESAVI onset time (1,671 days), whereas muscle twitching had the shortest mean onset time (13.39 days) (Table 3; Figures 4, 5).

**Table 3:** Onset time of ESAVIs for hepatitis B vaccine.

Group	N
0–30 days	53,466
121–150 days	318
151–180 days	256
181–360 days	733
31–60 days	1,443
61–90 days	726
91–120 days	432
>360 days	1,405

### 3.4 Multivariable logistic regression analysis

Table 4 summarizes the multivariable logistic regression models assessing factors associated with fatal outcomes (Death) in patients receiving treatment. Males had a significantly higher risk of death than females, which revealed an adjusted OR of 1.499 (95% CI = 1.323–1.699, *p* < 0.001), indicating that male patients are 49.9% more likely to experience fatal outcomes. Patients treated with other vaccines also demonstrated a higher likelihood of death, with an adjusted OR of 1.276 (95% CI = 1.107–1.475, *p* = 0.001). Conversely, patients between ages of 18 and 64 had a reduced risk of death, with an adjusted OR of 0.153 (95% CI = 0.123–1.188, *p* < 0.001).

**Table 4:** Multivariable logistic regression analysis of death for hepatitis B vaccine.

Characteristics	OR	95%CI	*P*-value
Age			
<18	Reference		
18–64	0.153	0.123–1.188	<0.001
≥65	1.286	0.859–1.850	0.196
Gender			
Female	Reference		
Male	1.499	1.323–1.699	<0.001
Vaccine alone			
Yes	Reference		
No	1.276	1.107–1.475	0.001

## 4 Discussion

Our study systematically reviewed nearly two decades of anonymized reports of hepatitis B vaccine within the VAERS database. This analysis aimed to offer a comprehensive overview of medications that potentially cause musculoskeletal-related adverse events. We compiled and analyzed data from 76,887 reports to summarize a list of the top 20 PTs most frequently associated with hepatitis B vaccine. Employing disproportionality analysis, we identified 5 PTs that significantly increase the risk of musculoskeletal-related adverse events. This selective approach not only provides robust data support for guiding individualized medication strategies for patients with HBV but also contributes valuable supplementary information for potential musculoskeletal adverse reactions to be included on vaccine labels.

Fibrosis tendinous exhibited the highest ROR value (ROR = 251.82) among all PTs, suggesting a strong association with hepatitis B vaccination. Tendon fibrosis, characterized by excessive extracellular matrix (ECM) deposition (13), may be driven by chronic inflammatory responses induced by vaccine adjuvants. Aluminum-based adjuvants, for instance, stimulate macrophages, dendritic cells, and Th2-type immune responses, triggering a chronic inflammatory cascade (12, 14, 15). This process may lead to aberrant tissue repair and fibrosis formation at local sites. While direct evidence linking tendon fibrosis to hepatitis B vaccination is currently unavailable, studies have reported that hepatitis B vaccination can exacerbate liver fibrosis (16). Furthermore, one study found that fibrosis significantly reduces antibody titers (17), indicating a possible interplay. The chronic nature of fibrosis tendinous aligns with its long onset time (occurring years post-vaccination) (18), potentially due to prolonged immune activation or an imbalance in tissue repair following localized damage.

Myofascitis and fasciitis displayed ROR values of 107.51 and 71.52, respectively, indicating significant muscle and fascial-related ESAVIs following hepatitis B vaccination. Studies suggest that aluminum adjuvants can persist at the injection site, forming “aluminum granulomas,” and induce macrophagic myofasciitis (MMF), leading to both local and systemic symptoms (19, 20). The prolonged latency of myofascitis supports the hypothesis that the slow-release properties of aluminum adjuvants play a critical role (21). Fasciitis may arise from an imbalance in the immune-inflammatory network triggered by vaccination (22). This condition can be caused by various factors, including mechanical overload (e.g., excessive plantar pressure), eosinophilic infiltration, macrophage and monocyte proliferation, degeneration, or bacterial infection (23).

Nuchal rigidity, with an ROR value of 16.19, represents a moderate signal. It often reflects acute inflammation of local muscles or fascia, potentially linked to short-term immune activation following vaccination. The rapid release of pro-inflammatory cytokines (e.g., IL-6, TNF-*α*) may cause localized muscle tension (24). A case report documented Isaac’s syndrome, characterized by peripheral nerve hyperexcitability, spontaneous muscle twitching, and stiffness, following HPV vaccination. This condition is typically associated with contactin-associated protein-like 2 (CASPR2) and leucine-rich glioma inactivated 1 (LGI1) antibodies (25). Temporal analysis revealed that the average onset time for nuchal rigidity was relatively short (6.993 days), supporting its primary mechanism as acute immune-inflammatory response rather than chronic changes (26).

Osteoarthritis showed an ROR value of 7.56, indicating a potential but weaker association with hepatitis B vaccination. Although osteoarthritis is typically a degenerative disease, vaccination may accelerate its progression through immune-mediated inflammatory processes (27). Brief but intense systemic inflammation post-vaccination could trigger oxidative stress and matrix degradation within joint tissues (28, 29). Individual susceptibility, such as a history of joint disease or genetic predisposition, might exacerbate this process. Research suggests that vaccination-induced activation of inflammatory pathways, including IL-1β and TNF-*α*, can induce chondrocyte apoptosis and overexpression of matrix metalloproteinases, thereby accelerating joint degeneration (30).

This study has certain limitations. First, the VAERS database is a spontaneous reporting system that relies on voluntarily submitted adverse event reports, which may result in underreporting and missing data. Second, the data in VAERS is reported voluntarily by healthcare providers, vaccine manufacturers, patients, and their families. Inaccurate descriptions may hinder the accurate assessment of ESAVI occurrence rates or the determination of causal relationships with vaccination. Furthermore, VAERS cases frequently lack complete information, such as dosage, comorbidities, onset times, and other essential details. Therefore, while the findings derived from data mining provide valuable insights, further evaluation and validation are necessary. This should involve the application of appropriate causal inference methods, integrating results from data mining, clinical trials, and real-world ESAVI cases.

## 5 Conclusion

These findings underscore the importance of monitoring and intervening in vaccine-related musculoskeletal ESAVIs. For individuals with a history of musculoskeletal or inflammatory diseases, such as arthritis, thorough pre-vaccination assessments and post-vaccination follow-ups may help reduce ESAVI incidence. Collecting comprehensive patient history is critical for optimizing vaccination safety. Furthermore, the development of safer and more effective adjuvants, along with mechanistic studies on vaccine-induced ESAVIs, should be prioritized in future research. Such efforts not only reduce the occurrence of adverse events but also bolster public confidence in vaccination programs.

## Data Availability

The datasets presented in this study can be found in online repositories. The names of the repository/repositories and accession number(s) can be found at: https://vaers.hhs.gov/.
