# Supplementary material for: Musculoskeletal adverse events reported post-hepatitis B vaccination in the vaccine adverse event reporting system
Source: Front Public Health. 2025 May 2;13:1560973. doi: 10.3389/fpubh.2025.1560973 (PMC12081253; doi:10.3389/fpubh.2025.1560973)
Supplement: Supplementary file 1 [file Table_1.docx]

Table S1 SOC distribution of HBV vaccine

| SOC | N | ROR(95%Cl) | PRR(χ^2^) | EBGM(EBGM05) | IC(IC025) |
| --- | --- | --- | --- | --- | --- |
| GASTROINTESTINAL DISORDERS | 18646 | 1.29 ( 1.27 - 1.31 ) | 1.27 ( 1072.09 ) | 1.26 ( 1.24 ) | 0.33 ( 0.31 ) |
| INFECTIONS AND INFESTATIONS | 11246 | 0.83 ( 0.82 - 0.85 ) | 0.84 ( 348.49 ) | 0.84 ( 0.83 ) | -0.24 ( -0.27 ) |
| GENERAL DISORDERS AND ADMINISTRATION SITE CONDITIONS | 71386 | 0.94 ( 0.93 - 0.94 ) | 0.95 ( 222.7 ) | 0.95 ( 0.95 ) | -0.07 ( -0.08 ) |
| SKIN AND SUBCUTANEOUS TISSUE DISORDERS | 25883 | 1.53 ( 1.51 - 1.55 ) | 1.48 ( 4114.06 ) | 1.46 ( 1.44 ) | 0.55 ( 0.53 ) |
| NERVOUS SYSTEM DISORDERS | 34306 | 1.09 ( 1.08 - 1.1 ) | 1.08 ( 212.46 ) | 1.08 ( 1.07 ) | 0.11 ( 0.09 ) |
| MUSCULOSKELETAL AND CONNECTIVE TISSUE DISORDERS | 16921 | 0.89 ( 0.87 - 0.9 ) | 0.89 ( 229.4 ) | 0.89 ( 0.88 ) | -0.16 ( -0.18 ) |
| INVESTIGATIONS | 32088 | 0.64 ( 0.63 - 0.64 ) | 0.68 ( 5822.5 ) | 0.68 ( 0.68 ) | -0.55 ( -0.57 ) |
| INJURY, POISONING AND PROCEDURAL COMPLICATIONS | 9379 | 0.79 ( 0.78 - 0.81 ) | 0.8 ( 485.24 ) | 0.8 ( 0.79 ) | -0.32 ( -0.35 ) |
| PSYCHIATRIC DISORDERS | 8839 | 1.77 ( 1.73 - 1.8 ) | 1.74 ( 2720.98 ) | 1.71 ( 1.68 ) | 0.77 ( 0.74 ) |
| BLOOD AND LYMPHATIC SYSTEM DISORDERS | 3051 | 1.18 ( 1.14 - 1.23 ) | 1.18 ( 83.05 ) | 1.18 ( 1.14 ) | 0.23 ( 0.18 ) |
| IMMUNE SYSTEM DISORDERS | 2331 | 1.67 ( 1.6 - 1.74 ) | 1.67 ( 597.62 ) | 1.64 ( 1.58 ) | 0.71 ( 0.65 ) |
| SOCIAL CIRCUMSTANCES | 497 | 0.44 ( 0.4 - 0.48 ) | 0.44 ( 346.29 ) | 0.45 ( 0.42 ) | -1.16 ( -1.29 ) |
| CONGENITAL, FAMILIAL AND GENETIC DISORDERS | 331 | 2.61 ( 2.34 - 2.92 ) | 2.61 ( 307.76 ) | 2.51 ( 2.28 ) | 1.33 ( 1.16 ) |
| EYE DISORDERS | 4204 | 1.18 ( 1.15 - 1.22 ) | 1.18 ( 112.47 ) | 1.17 ( 1.14 ) | 0.23 ( 0.19 ) |
| EAR AND LABYRINTH DISORDERS | 1328 | 0.56 ( 0.53 - 0.6 ) | 0.57 ( 437.04 ) | 0.57 ( 0.55 ) | -0.8 ( -0.88 ) |
| RESPIRATORY, THORACIC AND MEDIASTINAL DISORDERS | 8986 | 0.71 ( 0.69 - 0.72 ) | 0.72 ( 1022.39 ) | 0.72 ( 0.71 ) | -0.47 ( -0.5 ) |
| VASCULAR DISORDERS | 7648 | 1.75 ( 1.71 - 1.79 ) | 1.73 ( 2290.7 ) | 1.7 ( 1.67 ) | 0.76 ( 0.73 ) |
| RENAL AND URINARY DISORDERS | 1245 | 1.23 ( 1.16 - 1.3 ) | 1.23 ( 50.54 ) | 1.22 ( 1.16 ) | 0.29 ( 0.2 ) |
| NEOPLASMS BENIGN, MALIGNANT AND UNSPECIFIED (INCL CYSTS AND POLYPS) | 295 | 0.74 ( 0.66 - 0.83 ) | 0.74 ( 26.97 ) | 0.74 ( 0.67 ) | -0.43 ( -0.6 ) |
| HEPATOBILIARY DISORDERS | 1941 | 6.15 ( 5.86 - 6.45 ) | 6.12 ( 7168.6 ) | 5.41 ( 5.2 ) | 2.44 ( 2.37 ) |
| CARDIAC DISORDERS | 2082 | 0.36 ( 0.34 - 0.38 ) | 0.36 ( 2338.21 ) | 0.37 ( 0.36 ) | -1.43 ( -1.5 ) |
| SURGICAL AND MEDICAL PROCEDURES | 1702 | 0.5 ( 0.48 - 0.52 ) | 0.5 ( 836.74 ) | 0.51 ( 0.49 ) | -0.97 ( -1.04 ) |
| METABOLISM AND NUTRITION DISORDERS | 2209 | 1.14 ( 1.1 - 1.19 ) | 1.14 ( 38.71 ) | 1.14 ( 1.1 ) | 0.19 ( 0.13 ) |
| ENDOCRINE DISORDERS | 262 | 1.23 ( 1.09 - 1.39 ) | 1.23 ( 10.93 ) | 1.22 ( 1.1 ) | 0.29 ( 0.11 ) |
| REPRODUCTIVE SYSTEM AND BREAST DISORDERS | 357 | 0.13 ( 0.12 - 0.15 ) | 0.13 ( 2009.18 ) | 0.14 ( 0.13 ) | -2.87 ( -3.02 ) |
| PREGNANCY, PUERPERIUM AND PERINATAL CONDITIONS | 411 | 1 ( 0.91 - 1.1 ) | 1 ( 0 ) | 1 ( 0.92 ) | 0 ( -0.14 ) |
| PRODUCT ISSUES | 245 | 0.64 ( 0.56 - 0.72 ) | 0.64 ( 50.2 ) | 0.64 ( 0.58 ) | -0.64 ( -0.82 ) |
